# Occult Intraamniotic Infection at the Time of Midtrimester Genetic Amniocentesis: A Reassessment

**DOI:** 10.1155/S1064744994000530

**Published:** 1994

**Authors:** Peter H. Cherouny, Glenn A. Pankuch, John J. Botti

**Affiliations:** 1 Department of Obstetrics and Gynecology University of Vermont Burlington VT USA; 2 Department of Obstetrics and Gynecology Pennsylvania State University Hershey PA USA; 3 Department of Pathology Pennsylvania State University Hershey PA USA; 4 Medical Center Hospital of Vermont Shepardson 335 Burlington VT 05401 USA

## Abstract

*Objective:* The objective of this study was to reevaluate the incidence of occult early midtrimester intraamniotic infection in asymptomatic patients at the time of genetic amniocentesis.

*Methods:* A total of 177 amniotic fluid (AF) specimens from patients referred for genetic amniocentesis between 15 and 20 postmenstrual weeks were evaluated for the presence of bacteria by detailed light microscopy, after Gram and Wright stain, and by cultures for aerobic and anaerobic baceria, *Mycoplasma* sp., and *Ureaplasma urealyticum.* Seventy-seven AF specimens were also tested for the presence of bioactive leukoattractants by a leukotaxis bioassay.

*Results:* All fluids were negative for bacteria and bioactive leukoattractants [95% confidence interval (CI), 0–1.9%; 99% CI, 0–2.9%]. This is significantly less than a recently reported incidence of 5.09% (*P* = 0.002). Incidentally, artifacts with light microscopic morphology consistent with spermatozoa were found during the detailed light microscopic evaluation of AF Gram stains from 2 (1.1%) AF samples in otherwise uneventful pregnancies, a previously unreported finding. Scanning electron microscopy was used to confirm the light microscopic findings.

*Conclusions:* Occult intraamniotic infection in the second trimester is not as high as recently reported. AF culture in all cases of second-trimester amniocentesis is not necessary. The identification of spermatozoa on Gram stain of second-trimester AF specimens needs further confirmation.


Infectious Diseases in Obstetrics and Gynecology 2:136–139 (1994)
© 1994 Wiley-Liss, Inc.

### Occult Intraamniotic Infection at the Time of Midtrimester Genetic Amniocentesis: A Reassessment

**Peter H. Cherouny, Glenn A. Pankuch, and John J. Botti**

*Department of Obstetrics and Gynecology, University of Vermont, Burlington, VT (P.H.C.), and Departments of Obstetrics and Gynecology (J.J.B.) and Pathology (G.A.P.), Pennsylvania State University, Hershey, PA*

### Abstract

**Objective:** The objective of this study was to reevaluate the incidence of occult early midtrimester intraamniotic infection in asymptomatic patients at the time of genetic amniocentesis.

**Methods:** A total of 177 amniotic fluid (AF) specimens from patients referred for genetic amniocentesis between 15 and 20 postmenstrual weeks were evaluated for the presence of bacteria by detailed light microscopy, after Gram and Wright stain, and by cultures for aerobic and anaerobic baceria, *Mycoplasma* sp., and *Ureaplasma urealyticum*. Seventy-seven AF specimens were also tested for the presence of bioactive leukoattractants by a leukotaxis bioassay.

**Results:** All fluids were negative for bacteria and bioactive leukoattractants [95% confidence interval (CI), 0–1.9%; 99% CI, 0–2.9%]. This is significantly less than a recently reported incidence of 5.09% ($P = 0.002$). Incidentally, artifacts with light microscopic morphology consistent with spermatozoa were found during the detailed light microscopic evaluation of AF Gram stains from 2 (1.1%) AF samples in otherwise uneventful pregnancies, a previously unreported finding. Scanning electron microscopy was used to confirm the light microscopic findings.

**Conclusions:** Occult intraamniotic infection in the second trimester is not as high as recently reported. AF culture in all cases of second-trimester amniocentesis is not necessary. The identification of spermatozoa on Gram stain of second-trimester AF specimens needs further confirmation.
© 1994 Wiley-Liss, Inc.

Key words
Amniotic fluid, mycoplasmas, leukoattractants

It has been well established that bacteria and bioactive leukoattractants may be identified in amniotic fluid (AF) when chorioamniotic membranes are intact.[1,2] Positive AF cultures in patients in idiopathic preterm labor with intact membranes have a reported incidence of 4–26%. The most frequently isolated organisms in this setting include *Fusobacterium nucleatum*, *Mycoplasma sp.*, and *Ureaplasma urealyticum*. Positive AF cultures are almost universally associated with failed tocolysis and delivery soon after AF collection. The presence of AF leukoattractants determined by the leukotaxis bioassay is also a sensitive marker for AF infection, preterm delivery, and histologic chorioamnionitis in the preterm-labor population.[3] The leukotaxis assay is sensitive to a wide range and concentration of leukotactic factors and detects biologically active forms of leukoattractants. This is an advantage over immunologic assays of specific leukoattractants which identify immunoactive forms without addressing bioactivity.

The incidence of occult AF infection at the time of genetic amniocentesis in the early second trimester is not as well established. It has been recently

Address correspondence/reprint requests to Dr. Peter H. Cherouny, Medical Center Hospital of Vermont, Shepardson 335, Burlington, VT 05401.

Received February 15, 1994

Accepted July 1, 1994

Clinical Study reported as >5% (8/157), more than 10 times higher than the loss risk associated with amniocentesis.[4] The organisms identified in this previous report are not commonly isolated from AF collected from patients with preterm labor. Specifically, no anaerobic bacteria were identified and cultures for genital mycoplasms were not performed. The present study was, therefore, undertaken to reevaluate the incidence of occult infection of the amniotic cavity at the time of midtrimester genetic amniocentesis.

### SUBJECTS AND METHODS

AF was collected from 177 patients with viable singleton pregnancies, without identifiable abnormalities, referred for midtrimester amniocentesis between 15 and 20 weeks of gestation to University Hospital of the Pennsylvania State University School of Medicine. Informed consent was obtained in order to use an aliquot of AF for the study. The indications for amniocentesis included advanced maternal age (89%), low maternal serum alphafetoprotein (6%), parental anxiety (1%), and history of chromosomal abnormality in a prior pregnancy (4%). No patient related a history consistent with ruptured amniotic membranes and all had normal AF on ultrasound. Amniocentesis was performed by one of the authors (P.H.C. or J.J.B.), under ultrasound guidance and with strict asepsis, using a 20-gauge, 88.9-mm spinal needle.

AF was evaluated for bacteria by Gram stain, Wright stain, aerobic and anaerobic cultures, and culture for *Mycoplasma* sp. and *U. urealyticum*, as previously described.[2] In brief, AF was sent to the research laboratory for processing within 1 h of collection. When delays of >1 h but <12 h were anticipated, a portion of the sample was inoculated into a Port-A-Cul vial (BBL Microbiology Systems, Cockeysville, MD) for later culture and staining. The remaining fluid was refrigerated at 4°C until processed for *Mycoplasma* culture and, if fluid was available, for leukotaxis assay.

After Gram staining, AF was cultured for aerobic and anaerobic bacteria by spreading 0.1-, 0.01-, and 0.001-ml aliquots of uncentrifuged AF in duplicate on the following media: trypticase soy agar with 5% sheep blood (BBL Microbiology Systems); chocolate agar (BBL Microbiology Systems); prereduced chopped-meat glucose broth (Scott Laboratories, Fiskeville, RI); enriched brucella blood and laked blood agars (incubated anaerobically).

After culturing, AF was centrifuged at 1,000*g* for 10 min. After Wright staining, the pellet was cultured for *M. hominis* and *U. urealyticum* in the following manner. Ten microliters of the pellet was inoculated into urease color-test broth U9C. Three 10-fold serial dilutions were prepared in the U9C broth (incubated aerobically at 37°C for 21 days). Ten microliters from the pellet and 10 μl from each serial dilution were spread onto A8 differential agar medium (incubated anaerobically).

An aliquot of AF, available from 77 samples after culturing, was frozen at −70°C until evaluation for bioactive leukoattractants by the leukotaxis assay, as previously described.[3] No change in leukoattractant activity has been noted for samples stored in this manner for up to 2 years. Statistical analysis was performed using Fisher's exact test with confidence intervals (CIs) determined on percentages by Poisson distribution. A value of $P < 0.05$ was considered statistically significant.

### RESULTS

All bacteriologic studies were negative on all fluid specimens (0–1.9% and 0–2.9%, 95% and 99% CIs, respectively). In addition, no bioactive leukoattractants were identified in the AF aliquots available for testing with the leukotaxis bioassay. There were no immediate or delayed complications associated with amniocentesis. Specifically, no pregnancy losses occurred within 4 weeks of amniocentesis. Gram stain from 2 AF samples was observed to have artifacts with light microscopic morphologic characteristics consistent with spermatozoa, a finding not previously reported (Fig. 1). Scanning electron microscopy was used to confirm the presence of spermatozoa on the Gram-stained specimens (Figs. 2–4). These AF specimens did not contain bacteria or bioactive leukoattractants. Both fetuses were male.

### DISCUSSION

This study did not find bacterial colonization in the early second trimester in patients presenting for genetic amniocentesis. Furthermore, this study failed to show genital mycoplasmas or bioactive leukoattractants in any AF specimen tested in addition to negative aerobic and anaerobic AF cultures in asymptomatic patients. These data are in contra-

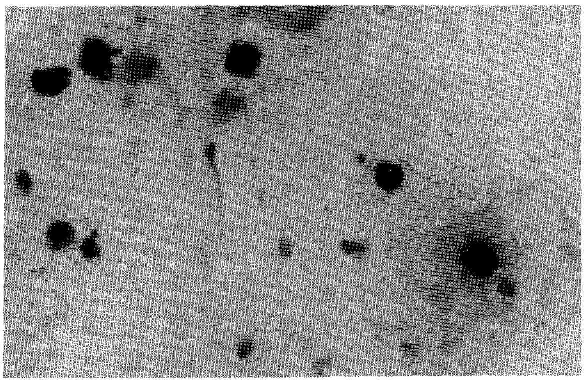

Fig. 1. Oil-immersion light microscopy of AF Gram stain revealing possible spermatozoa among cellular debris. ×1,000.

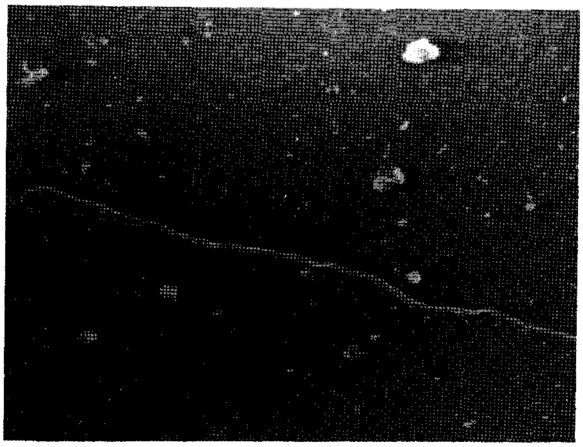

Fig. 2. Scanning electron microscopic morphology of spermatozoa identified on AF Gram stain from second-trimester genetic amniocentesis specimen. × 1,000.

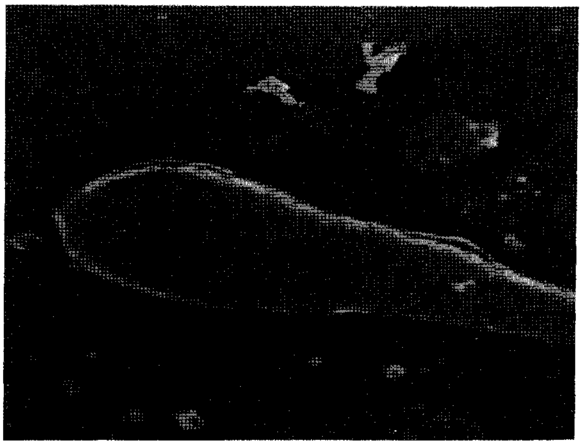

Fig. 3. Scanning electron photomicrograph. The head and neck regions of spermatozoa identified on AF Gram stain from second-trimester genetic amniocentesis specimen. ×5,000.

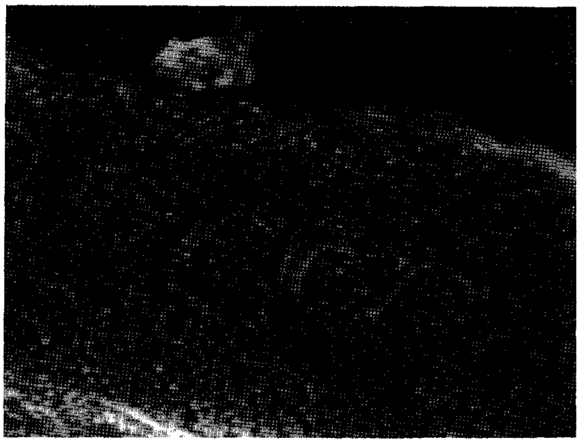

Fig. 4. Scanning electron photomicrograph. The mitochondrial cristae are seen in the center of the neck region of spermatozoa identified on AF Gram stain from second-trimester genetic amniocentesis specimen. ×50,000.

distinction to the recently published report of a 5.09% (8/157) incidence of occult AF infection at the time of second-trimester amniocentesis.[4] They do, however, agree with the findings of Thomsen et al.,[5] who reported culturing no *M. hominis* and had 1 positive culture for *U. urealyticum* of 150 midtrimester AF specimens.

Genital mycoplasmas are a common isolate in AF obtained from patients in preterm labor both in our laboratory and other laboratories.[1,3] Our prior successful culturing of *F. nucleatum* and the genital mycoplasmas in patients with preterm labor supports the accuracy of our culture results. The absence of AF bioactive leukoattractants, previously shown to be accurate predictors of preterm delivery, failed tocolysis, and histologic chorioamnionitis in the preterm labor/intact membrane population, provides additional supportive evidence that the negative cultures were accurate.

The high culture-positive rate and the failure to culture for *Mycoplasma* sp. and *U. urealyticum* in this prior report prompted the current study. In addition, the bacteria isolated by Goldstein et al.[4] from second-trimester AF were generally different from those cultured in reported studies on AF from patients in preterm labor with intact membranes.[1,3] A closer evaluation of the report by Goldstein et al.[4] reveals that 5 of the cultures showed only scant growth, defined as <10 colonies/plate. Moreover, whether the aborted pregnancy had scant growth of *Escherichia coli*, as reported in the text, or overabundant growth, as reported in the table, cannot be determined. The 3 positive cultures in the previous report with bacterial growth greater than scant would give an incidence of 3/157 (1%), not statistically different from that reported in the present study.

The documentation of an accurate incidence of occult infection in the second trimester is important when considering 1) the possible association with complications of amniocentesis and 2) the recommendation by Goldstein et al.[4] that bacteriologic evaluations be carried out on all AF specimens from the second trimester, at significant additional cost and without a recommendation of how to manage positive cultures in this setting. The data presented in this study do not support routine bacteriologic study of AF from patients presenting for genetic amniocentesis. The concordance of the bacteriologic tests and the AF samples evaluated for bioactive leukoattractants supports a low incidence of occult AF infection in the midtrimester. Whether the difference between the data previously reported and that presented in the current study is related to procedure or bacteriologic technique or patient population cannot be determined.

Of considerable interest is the previously unreported finding of spermatozoa on Gram stain of 2 second-trimester AF specimens from patients with intact membranes. Both patients delivered at term without complications. Sperm is a well-known carrier of bacteria;[6] and transmembranous movement of sperm, if confirmed, represents a potentially important etiology for intraamniotic infection. While scanning electron microscopy provides supportive evidence for this unexpected finding, the possibility that this finding represents artifacts of AF staining techniques cannot be entirely eliminated. Further studies are necessary to confirm or refute this potentially important finding.

### REFERENCES

1. Romero R, Sirtori M, Oyarzun E, et al.: Infection and labor. V. Prevalence, microbiology, and clinical significance of intraamniotic infection in women with preterm labor and intact membranes. Am J Obstet Gynecol 161: 817–824, 1989.
2. Pankuch GA, Cherouny PH, Botti JJ, Appelbaum PC: Amniotic fluid leukotaxis assay as an early indicator of chorioamnionitis. Am J Obstet Gynecol 161:802–807, 1989.
3. Cherouny PH, Pankuch GA, Botti JJ, Appelbaum PC: The presence of amniotic fluid leukoattractants accurately identifies histologic chorioamnionitis and predicts tocolytic efficacy in patients with idiopathic preterm labor. Am J Obstet Gynecol 167:683–688, 1992.
4. Goldstein I, Zimmer EZ, Merzbach D, Peretz BA, Paldi E: Intraamniotic infection in the very early phase of the second trimester. Am J Obstet Gynecol 163:1261–1263, 1990.
5. Thomsen AC, Taylor-Robinson D, Brogaard Hansen K, et al.: The infrequent occurrence of mycoplasmas in amniotic fluid from women with intact fetal membranes. Acta Obstet Gynecol Scand 63:425–429, 1984.
6. Toth A: Alternative causes of pelvic inflammatory disease. J Reprod Med 28:699–702, 1983.
